# Design, Rational, and Baseline Characteristics of the SONIC-HF Multicenter Registry

**DOI:** 10.31662/jmaj.2024-0364

**Published:** 2025-04-04

**Authors:** Nobuyuki Kagiyama, Kentaro Kamiya, Misako Toki, Hiroshi Saito, Kentaro Iwata, Yuya Matsue, Kenji Yoshioka, Kazuya Saito, Azusa Murata, Akihiro Hayashida, Junya Ako, Takeshi Kitai, Emi Maekawa

**Affiliations:** 1 Department of Cardiovascular Biology and Medicine, Juntendo University Graduate School of Medicine, Tokyo, Japan; 2 Department of Cardiology, The Sakakibara Heart Institute of Okayama, Okayama, Japan; 3 Department of Rehabilitation, School of Allied Health Sciences, Kitasato University, Sagamihara, Japan; 4 Department of Clinical Laboratory, The Sakakibara Heart Institute of Okayama, Okayama, Japan; 5 Department of Rehabilitation, Kameda Medical Center, Kamogawa, Japan; 6 Department of Rehabilitation, Kobe City Medical Center General Hospital, Kobe, Japan; 7 Department of Cardiology, Kameda Medical Center, Kamogawa, Japan; 8 Department of Rehabilitation, The Sakakibara Heart Institute of Okayama, Okayama, Japan; 9 Department of Cardiovascular Medicine, National Cerebral and Cardiovascular Centre, Osaka, Japan; 10 Department of Cardiovascular Medicine, Kitasato University School of Medicine, Sagamihara, Japan

**Keywords:** heart failure, skeletal muscle, ultrasound, frailty, sarcopenia

## Abstract

**Introduction:**

Skeletal muscle mass and function are crucial for assessing physical frailty, sarcopenia, and cachexia, which significantly impact the prognosis of geriatric patients with heart failure (HF). Ultrasound-based assessment of skeletal muscles offers a non-invasive, real-time alternative to traditional methods. The *compariSON of various methods In evaluatIon of sarCopenia in patients with Heart Failure* study (SONIC-HF) aimed to evaluate the feasibility and prognostic impact of ultrasound-based muscle assessment in geriatric patients with HF.

**Methods:**

This multicenter, prospective cohort study enrolled patients with HF aged ≥65 years who could ambulate independently at discharge. Certified observers assessed muscle thickness (biceps, quadriceps, rectus femoris, and diaphragm) using ultrasound at rest and during contraction. The primary endpoint was all-cause mortality. Secondary endpoints included HF hospitalization, unplanned hospital visits, and cardiovascular and non-cardiovascular mortality.

**Results:**

Of the 692 enrolled patients (median age 81 [interquartile range 74-86] years, 57.6% women, left ventricular ejection fraction 45% [32%-60%]), ultrasound-based muscle assessments were completed in 606 patients. Interobserver reliability was excellent (intraclass correlation coefficient 0.84-0.99). Median muscle thicknesses at rest and during contraction were: diaphragm 1.9 (1.6-2.3) mm and 2.9 (2.3-3.8) mm; biceps 19.6 (15.9-23.1) mm and 25.3 (21.3-29.5) mm; quadriceps 19.0 (15.0-23.5) mm and 24.8 (19.9-29.5) mm; rectus femoris 9.7 (7.1-12.3) mm and 12.1 (9.6-15.0) mm. The median follow-up time was 733.5 (438-882) days.

**Conclusions:**

The SONIC-HF registry will provide valuable insights into the feasibility and prognostic implications of ultrasound-based muscle assessment in geriatric patients with HF.

## Introduction

Heart failure (HF) is a prevalent and severe condition affecting approximately 26 million people worldwide ^(1)^. Due to the global aging trend, the number of patients with HF continues to rise. Notably, the population aged 65 and over is growing faster than other age groups. By 2050, one in six people globally will be over age 65 (16%), a rise from one in 11 in 2019 (9%) ^(2)^.

Muscle weakness is a recognized problem in both the general population and patients with HF. HF itself predisposes patients to muscle weakness due to factors like inflammation, inactivity, oxidative stress, malnutrition, and muscle cell death ^(3)^. In our previous research called FRAGILE-HF (prevalence and prognostic value of physical and social frailty in geriatric patients hospitalized for HF), we showed that physical frailty ^(4), (5)^, sarcopenia ^(6)^, and cachexia ^(2), (7)^ were highly prevalent among geriatric patients with HF and were significantly associated with increased adverse events. The assessments of muscle mass and function in FRAGILE-HF relied on tools like questionnaires, body and limb measurements, handgrip strength tests, and bioelectrical impedance analysis. However, these traditional methods have limitations, particularly in patients with HF with edema or other conditions that can obscure accurate assessments of muscle mass.

Ultrasound has emerged as a valuable tool for assessing skeletal muscle mass and function due to its non-invasive nature, real-time imaging capability, and accessibility. Compared with X-rays or magnetic resonance imaging (MRI), ultrasound is more practical for repeated assessments, particularly at the bedside. Additionally, ultrasound enables measurements of muscle thickness both at rest and during contraction, allowing for a dynamic evaluation of muscle function. Despite these advantages, its feasibility and prognostic value have not been extensively studied for patients with HF in multicenter settings.

To address this gap, the SONIC-HF study (*compariSON of various methods In evaluatIon of sarCopenia in patients with Heart Failure*) was initiated to evaluate the feasibility and prognostic impact of ultrasound-based skeletal muscle assessments in this high-risk population. In this paper, we summarize the design and rationale of the study and demonstrate the baseline characteristics of enrolled patients.

## Materials and Methods

### Study design

The SONIC-HF study was a multicenter, prospective cohort study conducted across four hospitals in Japan, aimed at evaluating the prevalence of muscle atrophy and its prognostic implications in geriatric patients with HF. The study primarily assessed muscle mass and function using ultrasound, supplemented by anthropometric measurements. The first and last patients were enrolled on January 30, 2018, and December 28, 2019, respectively. The protocol followed the Declaration of Helsinki and received approval from the institutional review boards of all participating centers. All participants were fully informed about the study’s objectives and procedures through an opt-out method and were assured of their right to withdraw at any time. As this was an observational study that did not entail invasive procedures or interventions, written informed consent was not required under the Ethical Guidelines for Medical and Health Research Involving Human Subjects, as per the Japanese Ministry of Health, Labour, and Welfare.

Comprehensive details of the study, including its objectives, eligibility criteria, primary outcomes, and participating hospitals, were registered and made publicly accessible on the University Hospital Information Network (UMIN-CTR, unique identifier: UMIN000031635) before the enrollment of the first participant. Although the study originally planned to involve six hospitals, logistical challenges prevented two centers from enrolling patients.

Eligible patients were those aged ≥65 years, admitted for decompensated HF, and able to ambulate independently at discharge. The study included only the first hospitalization for each patient during the study period, with HF decompensation diagnosed according to the Framingham criteria ^(8)^. Patients were excluded if they had undergone heart transplantation, were using a left ventricular assist device, were receiving chronic peritoneal dialysis or hemodialysis, or were diagnosed with acute myocarditis. Additionally, patients were excluded if they lacked B-type natriuretic peptide (BNP) or N-terminal-proBNP (NT-proBNP) data or had low levels of BNP (<100 pg/mL) or NT-proBNP (<300 pg/mL) at admission. The study cohort included patients with HF with reduced, mildly reduced, and preserved ejection fraction.

### Data acquisition

Patient characteristics, including demographic and laboratory data, physical function, medication prescriptions, and ultrasound-based muscle measurements, were collected prospectively once the patient had stabilized following the completion of intravenous drug administration. Echocardiography was also performed before discharge, in accordance with published guidelines ^(9)^. Significant valvular heart diseases were defined as moderate or severe valvular heart disease ^(10)^.

**Figure 1.**
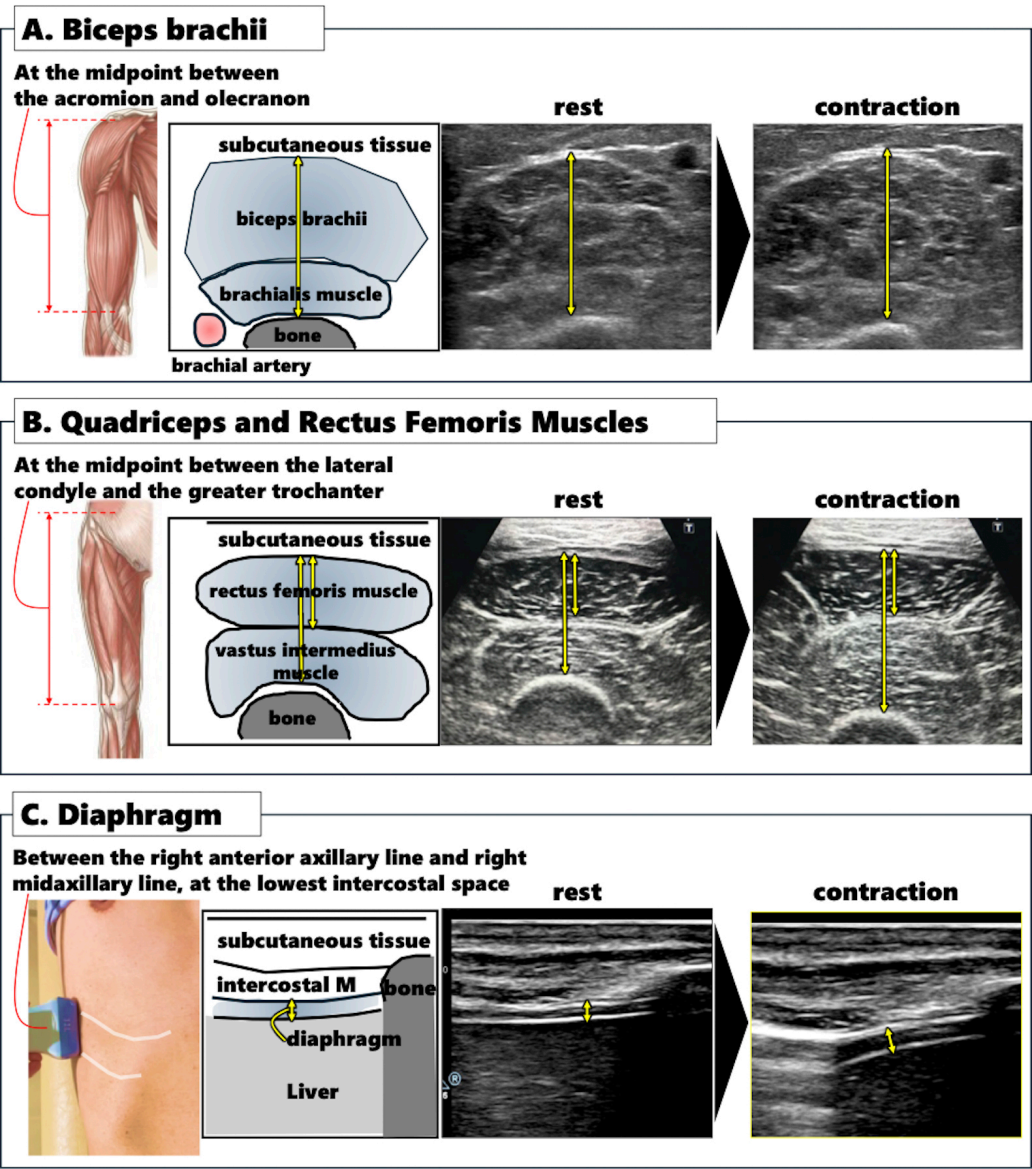
Ultrasound assessment of skeletal muscles. Panel A: Measurement of the biceps brachii and brachialis thickness at the midpoint between the acromion and olecranon, with the patient seated or lying down. Panel B: Quadriceps and rectus femoris muscle thickness measured at the midpoint between the lateral condyle and the greater trochanter, with the patient in a supine position. Panel C: Diaphragm thickness measured between the right anterior and mid-axillary lines at the lowest intercostal space, during both natural expiration and full inspiration.

To assess muscle thickness, specific anatomical sites were evaluated. The midpoint between the acromion and olecranon was used to measure the thickness of the biceps brachii and brachialis muscles, with the patient either seated in a chair or lying on a bed (Figure 1A). The thickness of the quadriceps and rectus femoris muscles was measured at the midpoint between the lateral condyle and the greater trochanter, with the patient in a supine position (Figure 1B). A linear probe with a frequency range of 7.5-13.0 MHz was placed perpendicular to the tissue interface, and the distance from the adipose tissue-muscle interface to the muscle-bone interface was recorded as muscle thickness ^(11), (12)^. Muscle contraction was evaluated using isotonic and isometric exercises, with the upper limbs assessed during arm bending and the lower limbs during leg extension without bending ^(11), (12)^. Diaphragm thickness was measured between the right anterior and mid-axillary lines at the lowest intercostal space using the same probe as for the upper and lower limb measurements (Figure 1C) ^(13)^. These measurements were performed twice during both natural expiration (at rest) and full inspiration, with the mean value of the two measurements used for analysis.

Since many observers were initially unfamiliar with the measurement procedure, a certification program for ultrasound scanning was implemented. Observers practiced scanning on volunteers and submitted images to the core lab for feedback. Only after the core lab certified that the observer could reliably measure muscles without significant errors, they were permitted to scan actual patients. All measurements were conducted twice, both at rest and during contraction, with the mean value of the two measurements used for analysis.

Interobserver variability was assessed by two blinded observers who independently measured the same randomly selected images twice. The number of images used for this analysis ranged from 40 to 46 for each muscle and phase. The average values from each observer were compared, and the level of agreement between them was determined to evaluate interobserver reliability.

### End points

The primary outcome predefined in this study was all-cause mortality. After discharge, most patients had follow-up appointments at outpatient clinics at least every 3 months, with additional visits as required by their individual medical needs. For patients who did not attend these clinics, outcome data were obtained via telephone interviews, either by reviewing medical records from other healthcare facilities or by speaking with the patient’s family. Causes of death were categorized as either cardiovascular or non-cardiovascular, following established definitions. Cardiovascular deaths included those resulting from HF, acute coronary syndrome, stroke, ventricular arrhythmias, and other cardiovascular diseases. Sudden death from an unknown cause was also classified as cardiovascular death ^(14)^.

Secondary outcomes included HF hospitalization and unplanned hospital visits due to HF exacerbation. HF hospitalization was defined as an unplanned admission with a primary diagnosis of HF lasting at least 24 hours (or spanning calendar days), accompanied by clear new onset or worsening symptoms, and requiring the initiation of new HF treatments or intensification of existing HF therapy during the hospitalization. An unplanned visit due to HF exacerbation was defined as an unscheduled visit to a clinic, hospital, or emergency department (ED), where the primary diagnosis was worsening HF, with clear new onset or worsening of HF symptoms, requiring the initiation of new HF treatments or intensification of existing therapy in the ED or outpatient setting.

### Statistical Analysis

Continuous variables are presented as mean ± standard deviation or as medians with interquartile ranges (IQR) (1st and 3rd quartiles), depending on their distribution. Categorical variables are expressed as frequencies and percentages. To adjust for the association between the variable of interest and outcomes, including all-cause mortality and the composite of HF hospitalization and all-cause mortality, we employed the Meta-analysis Global Group in Chronic Heart Failure (MAGGIC) risk score ^(15)^ and BNP levels as covariates. The MAGGIC risk score has been well-validated for predicting outcomes in Japanese patients with HF ^(16), (17)^, and the inclusion of BNP levels at discharge has been shown to improve the score’s discrimination while maintaining adequate calibration ^(17)^.

## Results

### Population

A total of 692 patients were enrolled in the SONIC-HF study across four participating hospitals in Japan. Table 1 summarizes the patient characteristics. The median age of the cohort was 81 years (74-86 years), with 42.4% men and 57.6% women. The median body mass index was 21.1 kg/m^2^ (19.0-23.7 kg/m^2^), and the median body surface area was 1.51 m^2^ (1.37-1.64 m^2^). The median systolic blood pressure at admission was 112.0 mmHg (102.0-124.8 mmHg), and the median diastolic blood pressure was 61.0 mmHg (56.0-68.0 mmHg). The median heart rate was 70 bpm (61-80 bpm). Regarding the New York Heart Association (NYHA) classification, 19.9% of the patients were classified as NYHA class III-IV at discharge, indicating a significant proportion of patients with advanced symptoms even after completion of intensive HF management.

**Table 1.** Patient Characteristics.

	N	Median (IQR); n (%)
Age, years	692	81.0 (74.0 - 86.0)
Male	691	293 (42.4%)
Current smoker	690	62 (9.0%)
Body mass index, kg/m^2^	627	21.1 (19.0 - 23.7)
Body surface area, m^2^	627	1.51 (1.37 - 1.64)
Systolic blood pressure, mmHg	690	112.0 (102.0 - 124.8)
Diastolic blood pressure, mmHg	690	61.0 (56.0 - 68.0)
Heart rate, bpm	690	70 (61 - 80)
NYHA class III-IV	685	136 (19.9%)
Past medical history		
Previous HF hospitalization	691	351 (50.8%)
Hypertension	691	442 (64.0%)
Diabetes mellitus	691	285 (41.2%)
Dyslipidemia	690	247 (35.8%)
Peripheral artery disease	690	49 (7.1%)
Atrial fibrillation	691	332 (48.0%)
Chronic obstructive pulmonary disease	691	71 (10.3%)
Stroke or transient ischemic attack	691	80 (11.6%)
Coronary artery disease	690	264 (38.3%)
Cirrhosis	691	13 (1.9%)
Malignancy	691	84 (12.2%)
Echocardiography		
LVEF, %	647	45.0 (31.9 - 60.0)
LVEF categories, %	647	
HFpEF		272 (42.0%)
HFmrEF		104 (16.1%)
HFrEF		271 (41.9%)
Significant mitral regurgitation	646	287 (44.4%)
Significant aortic regurgitation	642	112 (17.4%)
Significant aortic stenosis	628	56 (8.9%)
Prescriptions		
Loop diuretics	691	590 (85.4%)
Tolvaptan	691	226 (32.7%)
ACE inhibitors or ARBs	691	386 (55.9%)
Beta-blockers	691	447 (64.7%)
MRAs	691	319 (46.2%)
Digitalis	691	23 (3.3%)
Laboratory data		
Hemoglobin, g/dL	689	11.5 (10.3 - 13.1)
Creatinine, mg/dL	692	1.21 (0.96 - 1.72)
eGFR, mL/min/1.73m^2^	691	45.8 (29.7 - 63.4)
Blood urea nitrogen, mg/dL	692	27.0 (20.0 - 38.8)
Albumin, g/dL	655	3.50 (3.10 - 3.80)
Sodium, mmol/L	692	139.0 (136.0 - 141.0)
Potassium, mmol/L	692	4.3 (4.0 - 4.7)
Hemoglobin A1c, %	465	6.0 (5.7 - 6.7)
BNP, pg/mL	531	235 (122 - 481)
NT pro-BNP, pg/mL	126	2,168 (900 - 3,306)
MAGGIC score	584	27.0 (23.0 - 31.0)

BMI, body mass index; BP, blood pressure; BNP, B-type natriuretic peptide; NYHA, New York Heart Association; COPD, chronic obstructive lung disease; ACE-I, angiotensin-converting enzyme inhibitors; ARB, angiotensin II receptor blockers; MRA, mineralocorticoid receptor antagonist

The cohort exhibited a high prevalence of comorbid conditions, which is typical for this demographic. A previous history of HF hospitalization was present in 50.8% of patients, and hypertension was the most common comorbidity, affecting 64.0% of the cohort, followed by atrial fibrillation (48.0%), diabetes mellitus (41.2%), and dyslipidemia (35.8%). Other notable comorbidities included coronary artery disease (38.3%), stroke or transient ischemic attack (11.6%), and chronic obstructive pulmonary disease (10.3%).

Echocardiographic assessments revealed a median left ventricular ejection fraction (LVEF) of 45.0% (31.9%-60.0%). The distribution of LVEF categories was relatively balanced: 42.0% of patients had HF with preserved EF (HFpEF), 41.9% had HF with reduced EF (HFrEF), and 16.1% had HF with mildly reduced EF (HFmrEF). Additionally, significant mitral regurgitation was present in 44.4% of patients, while significant aortic regurgitation and aortic stenosis were observed in 17.4% and 8.9% of patients, respectively.

In terms of pharmacotherapy and laboratory data, loop diuretics were prescribed to 85.4% of the cohort. Other frequently prescribed medications included beta-blockers (64.7%), angiotensin-converting enzyme inhibitors or angiotensin II receptor blockers (55.9%), and mineralocorticoid receptor antagonists (46.2%). The median hemoglobin level was 11.5 g/dL (10.3-13.1 g/dL), and the median creatinine level was 1.21 mg/dL (0.96-1.72 mg/dL), resulting in an estimated glomerular filtration rate of 45.8 mL/min/1.73 m^2^ (29.7-63.4 mL/min/1.73 m^2^). The median BNP level was 235 pg/mL (122-481 pg/mL), and the median NT-proBNP level was 2,168 pg/mL (900-3,306 pg/mL). The MAGGIC risk score had a median value of 27.0 (23.0-31.0).

### Ultrasound-based muscle assessment

Ultrasound-based skeletal muscle assessments were successfully conducted in 606 of the 692 patients initially enrolled in the SONIC-HF study. The difference in sample size was primarily due to patients being discharged early or unexpectedly, before completing the ultrasound examinations.

Interobserver variability was rigorously assessed to ensure the reliability of the ultrasound measurements, with intraclass correlation coefficients (ICCs) calculated for each muscle group. The biceps brachii showed an ICC of 0.989 (0.978-0.995) at rest, indicating high consistency, and 0.973 (0.948-0.986) during contraction. Similarly, the quadriceps demonstrated an ICC of 0.979 (0.963-0.988) at rest and 0.997 (0.994-0.998) during contraction, suggesting excellent reproducibility. The rectus femoris muscle had slightly lower but still robust ICC values, with 0.883 (0.798-0.934) at rest and 0.949 (0.909-0.971) during contraction. Diaphragm measurements, while showing lower ICCs, were still within acceptable ranges, with an ICC of 0.805 (0.669-0.889) at rest and 0.799 (0.653-0.888) during inspiration.

The median muscle thicknesses observed across the cohort are summarized in Table 2. Specifically, the biceps brachii had a thickness of 19.6 mm (15.9-23.1 mm) at rest and 25.3 mm (21.3-29.5 mm) during contraction. For the quadriceps, the thickness was 9.7 mm (7.1-12.3 mm) at rest and increased to 12.1 mm (9.6-15.0 mm) during contraction. The rectus femoris had a median thickness of 19.0 mm (15.0-23.5 mm) at rest, which increased to 24.8 mm (19.9-29.5 mm) during contraction. Finally, the diaphragm, a key respiratory muscle, had a median thickness of 1.88 mm (1.55-2.30 mm) at rest and 2.90 mm (2.25-3.75 mm) during inspiration.

**Table 2.** Ultrasound-Based Skeletal Muscle Thickness.

	N	Median (IQR); n (%)
Bicep thickness at rest, mm	606	19.6 (15.9 - 23.1)
Bicep thickness during contraction, mm	604	25.3 (21.3 - 29.5)
Rectus femoris thickness at rest, mm	604	9.7 (7.1 - 12.3)
Rectus femoris thickness during contraction, mm	603	12.1 (9.6 - 15.0)
Quadriceps thickness at rest, mm	606	19.0 (15.0 - 23.5)
Quadriceps thickness during contraction, mm	605	24.8 (19.9 - 29.5)
Diaphragm thickness at rest, mm	605	1.88 (1.55 - 2.30)
Diaphragm thickness during inspiration, mm	606	2.90 (2.25 - 3.75)

The study has completed its follow-up period, with a median follow-up duration of 733.5 (438.0-882.0) days, and survival analyses are set to be performed.

## Discussion

As the average age of patients with HF increases worldwide, comorbidities associated with HF are becoming more prevalent. Physical frailty, sarcopenia, and cachexia are particularly common comorbidities, especially in geriatric patients with HF. The SONIC-HF registry focuses on assessing muscle mass, a cornerstone in evaluating physical frailty, sarcopenia, and cachexia in patients with HF aged ≥65 years, with the aim of validating simple and widely applicable ultrasound-based techniques.

Mass and function are two key aspects of skeletal muscles and form the core of the pathophysiology of physical frailty and sarcopenia. Physical frailty is most often defined by five components: weight loss, exhaustion, low physical activity, slowness, and weakness ^(18)^, while sarcopenia is characterized by muscle strength, muscle mass, and physical performance ^(19), (20)^. Cachexia is defined by weight loss, decreased muscle strength, fatigue, anorexia, low fat-free mass, and abnormal biochemistry ^(2), (21)^. These comorbidities are common and significantly impact mortality and quality of life in patients with HF, and accordingly, it is highly recommended to assess these conditions in geriatric patients with HF ^(8), (22), (23)^. However, in clinical practice, the gold-standard methods for assessing muscle mass and function―dual-energy X-ray absorptiometry, MRI, or computed tomography―are rarely performed. Bioelectrical impedance analysis is easier but lacks sufficient accuracy, particularly in patients with HF who tend to have edema and increased extracellular water ^(24)^. Therefore, there is a strong need for simpler and more widely applicable methods to accurately assess muscle mass and function.

Ultrasound is widely employed in the medical assessment of various organs. It operates by emitting high-frequency sound waves, which are reflected off tissues and processed to generate detailed images. Skeletal muscles are well-suited for ultrasound assessment because they are located near the body’s surface, allowing for clear visualization with minimal interference from air or bone. Additionally, tissues close to the probe are imaged with greater clarity than deeper structures due to reduced signal dissipation. Furthermore, advances in ultrasound technology have led to the development of portable, handheld devices, which are now commonly used in clinical practice and are increasingly regarded as an extension of the physical examination.

Owing to these advantages, ultrasound has been increasingly used in the assessment of skeletal muscles. Sanada et al. ^(25)^ validated the accuracy of ultrasound-based muscle mass assessment in the intensive care setting, comparing it with MRI in 72 participants, and found strong correlations between muscle mass measured by MRI and muscle thickness measured by ultrasound. Ogawa et al. ^(26)^ investigated the reproducibility of ultrasound assessment of quadriceps thickness, reporting similar ICCs of 0.96-0.99. Watanabe et al. ^(27)^ demonstrated that quadriceps muscle thickness decreases with age in healthy Japanese individuals, with mean values of 25.9 ± 5.7 mm for men and 20.7 ± 4.8 mm for women aged ≥ 65 years. The median quadriceps thickness in our cohort was 20.1 mm (IQR 19.2-20.9 mm) at rest, which was thinner than in the above studies but aligned with previous findings that reported a median value of 20.1 mm (IQR 19.2-20.9 mm) in patients with HFpEF ^(28)^. Other studies have also demonstrated the association between skeletal muscle thickness and exercise capacity in patients with HF ^(29), (30), (31)^. Although these studies establish reliability and associations with muscle mass as measured by gold-standard methods, the prognostic value of ultrasound-based muscle thickness in patients with HF remains underexplored. Our multicenter registry will contribute significant insights into the literature regarding the prognostic value of these measurements.

Our study has several limitations. First, this registry does not employ core-laboratory analysis of ultrasound images, and the ultrasound equipment used is not standardized. As a result, ultrasound intensity was not measured in this study. The intensity of ultrasound signals from skeletal muscles may provide valuable information beyond muscle thickness ^(32)^. However, this requires offline analysis, which is not available on the machines used and significantly limits its clinical application. Therefore, we focused on muscle thickness, which is easier to measure at the bedside. Additionally, the study was conducted at only four sites and included only Japanese patients. Since body surface area and body mass index in Japanese patients differ significantly from those in Western populations, caution is needed when generalizing these findings to Western populations. Furthermore, the study exclusively enrolled patients aged ≥65 years, and those who passed away during HF hospitalization were not included. These factors might limit the generalizability of the findings and introduce selection bias.

In conclusion, the SONIC-HF registry has included 692 patients with geriatric patients with HF in Japan and measured ultrasound-based skeletal muscle thickness for 606 patients. The study tracked patients for 2 years and has detailed baseline and follow-up information. This will be the first and largest database of ultrasound-based skeletal muscle thickness for patients with HF and will uncover the clinical usefulness of this easy, reproducible, and widely applicable method of muscle assessment for patients with HF.

## Article Information

### Conflicts of Interest

Nobuyuki Kagiyama was affiliated with a department endowed by grants Paramount Bed, and received research grants from EchoNous. Inc. and AMI Inc., and received an honorarium from Novartis Japan, Otsuka Pharma, Eli Lilly, and Nippon Boehringer Ingelheim outside the submitted work. Yuya Matsue received an honorarium from Otsuka Pharmaceutical Co., Novartis Japan, AstraZeneca K.K., Ono Pharmaceutical Co., Ltd., Kyowa Kirin Co., Ltd., Bayer Japan, and Pfizer, Inc., and research funding outside the submitted work from Nippon Boehringer Ingelheim Co., Ltd., Pfizer Inc., Otsuka Pharmaceutical Co., EN Otsuka Pharmaceutical Co., Ltd., and Roche Diagnostics Japan. Kentaro Kamiya received funding outside the submitted work from Eiken Chemical Co., Ltd. and SoftBank Cor. Ltd. The other authors have no conflicts of interest to declare.

### Sources of Funding

This study was partially supported by the Novartis Foundation (Japan) for the Promotion of Health and by the Japan Society for the Promotion of Science KAKENHI (grant number 19K11424).

### Author Contributions

Nobuyuki Kagiyama: data acquisition, drafting the manuscript, statistical analysis, conceptualization. Kentaro Kamiya: data acquisition, critical review of the manuscript, conceptualization. Misako Toki: data acquisition, critical review of the manuscript. Hiroshi Saito: data acquisition, critical review of the manuscript. Kentaro Iwata: data acquisition, critical review of the manuscript. Yuya Matsue: data acquisition, critical review of the manuscript. Kenji Yoshioka: data acquisition, critical review of the manuscript. Kazuya Saito: data acquisition, critical review of the manuscript. Azusa Murata: data acquisition, critical review of the manuscript. Akihiro Hayashida: data acquisition, critical review of the manuscript. Junya Ako: data acquisition, critical review of the manuscript, supervision. Takeshi Kitai: data acquisition, critical review of the manuscript. Emi Maekawa: data acquisition, critical review of the manuscript, conceptualization.

## References

[1] Ambrosy AP, Fonarow GC, Butler J, et al. The global health and economic burden of hospitalizations for heart failure: lessons learned from hospitalized heart failure registries. J Am Coll Cardiol. 2014;63(12):1123-33. 24491689 10.1016/j.jacc.2013.11.053

[2] Maekawa E, Noda T, Maeda D, et al. Prognostic impact of cachexia by multi-assessment in older adults with heart failure: FRAGILE-HF cohort study. J Cachexia Sarcopenia Muscle. 2023;14(5):2143-51. 37434419 10.1002/jcsm.13291 PMC10570094

[3] Bozkurt B, Fonarow GC, Goldberg LR, et al. Cardiac rehabilitation for patients with heart failure: JACC expert panel. J Am Coll Cardiol. 2021;77(11):1454-69. 33736829 10.1016/j.jacc.2021.01.030

[4] Matsue Y, Kamiya K, Saito H, et al. Prevalence and prognostic impact of the coexistence of multiple frailty domains in elderly patients with heart failure: the FRAGILE-HF cohort study. Eur J Heart Fail. 2020;22(11):2112-9. 32500539 10.1002/ejhf.1926

[5] Nozaki K, Kamiya K, Hamazaki N, et al. Validity and utility of the questionnaire-based FRAIL scale in older patients with heart failure: findings from the FRAGILE-HF. J Am Med Dir Assoc. 2021;22(8):1621-1626.e2. 33785309 10.1016/j.jamda.2021.02.025

[6] Konishi M, Kagiyama N, Kamiya K, et al. Impact of sarcopenia on prognosis in patients with heart failure with reduced and preserved ejection fraction. Eur J Prev Cardiol. 2021;28(9):1022-9. 33624112 10.1093/eurjpc/zwaa117

[7] Fujimoto Y, Maeda D, Kagiyama N, et al. Prevalence and prognostic impact of the coexistence of cachexia and sarcopenia in older patients with heart failure. Int J Cardiol. 381;381:45-51. 36934990 10.1016/j.ijcard.2023.03.035

[8] Tsutsui H, Isobe M, Ito H, et al. JCS 2017/JHFS 2017 guideline on diagnosis and treatment of acute and chronic heart failure - digest version. Circ J. 2019;83(10):2084-184. 31511439 10.1253/circj.CJ-19-0342

[9] Tseng AS, Lopez-Jimenez F, Pellikka PA. Future guidelines for artificial intelligence in echocardiography. J Am Soc Echocardiogr. 2022;35(8):878-82. 35472568 10.1016/j.echo.2022.04.005

[10] Otto CM, Nishimura RA, Bonow RO, et al. 2020 ACC/AHA guideline for the management of patients with valvular heart disease: a report of the American College of Cardiology/American Heart Association joint committee on clinical practice guidelines. J Am Coll Cardiol. 2021;77:e25-197. 33342586 10.1016/j.jacc.2020.11.018

[11] Ogawa M, Mitsukawa N, Bemben MG, et al. Ultrasound assessment of adductor muscle size using muscle thickness of the thigh. J Sport Rehabil. 2012;21(3):244-8. 22713209 10.1123/jsr.21.3.244

[12] Takai Y, Ohta M, Akagi R, et al. Applicability of ultrasound muscle thickness measurements for predicting fat-free mass in elderly population. J Nutr Health Aging. 2014;18(6):579-85.24950147 10.1007/s12603-013-0419-7

[13] Cohn D, Benditt JO, Eveloff S, et al. Diaphragm thickening during inspiration. J Appl Physiol (1985). 1997;83(1):291-6. 9216975 10.1152/jappl.1997.83.1.291

[14] Kitai T, Miyakoshi C, Morimoto T, et al. Mode of death among Japanese adults with heart failure with preserved, midrange, and reduced ejection fraction. JAMA Netw Open. 2020;3(5):e204296. 32379331 10.1001/jamanetworkopen.2020.4296 PMC7206504

[15] Pocock SJ, Ariti CA, McMurray JJV, et al. Predicting survival in heart failure: a risk score based on 39 372 patients from 30 studies. Eur Heart J. 2013;34(19):1404-13. 23095984 10.1093/eurheartj/ehs337

[16] Yamaguchi T, Kitai T, Miyamoto T, et al. Effect of optimizing guideline-directed medical therapy before discharge on mortality and heart failure readmission in patients hospitalized with heart failure with reduced ejection fraction. Am J Cardiol. 2018;121(8):969-74. 29477488 10.1016/j.amjcard.2018.01.006

[17] Sawano M, Shiraishi Y, Kohsaka S, et al. Performance of the MAGGIC heart failure risk score and its modification with the addition of discharge natriuretic peptides. ESC Heart Fail. 2018;5(4):610-9. 29520978 10.1002/ehf2.12278 PMC6073038

[18] Fried LP, Tangen CM, Walston J, et al. Frailty in older adults: evidence for a phenotype. J Gerontol A Biol Sci Med Sci. 2001;56(3):M146-56. 11253156 10.1093/gerona/56.3.m146

[19] Chen LK, Woo J, Assantachai P, et al. Asian Working Group for Sarcopenia: 2019 Consensus update on sarcopenia diagnosis and treatment. J Am Med Dir Assoc. 2020;21(3):300-7.e2. 32033882 10.1016/j.jamda.2019.12.012

[20] Cruz-Jentoft AJ, Bahat G, Bauer J, et al. Sarcopenia: revised European consensus on definition and diagnosis. Age Ageing. 2019;48(1):16-31. 30312372 10.1093/ageing/afy169 PMC6322506

[21] Evans WJ, Morley JE, Argilés J, et al. Cachexia: a new definition. Clin Nutr. 2008;27(6):793-9. 18718696 10.1016/j.clnu.2008.06.013

[22] Kida K, Miyajima I, Suzuki N, et al. Nutritional management of heart failure. J Cardiol. 2023;81(3):283-91. 36370995 10.1016/j.jjcc.2022.11.001

[23] Sato K, Kamiya K, Hamazaki N, et al. Association of sarcopenia defined by different skeletal muscle mass measurements with prognosis and quality of life in older patients with heart failure. J Cardiol. 2024;84(1):59-64. 38135146 10.1016/j.jjcc.2023.12.003

[24] Saito H, Matsue Y, Maeda D, et al. Prognostic values of muscle mass assessed by dual-energy x-ray absorptiometry and bioelectrical impedance analysis in older patients with heart failure. Geriatr Gerontol Int. 2022;22(8):610-5. 35751442 10.1111/ggi.14424

[25] Sanada K, Kearns CF, Midorikawa T, et al. Prediction and validation of total and regional skeletal muscle mass by ultrasound in Japanese adults. Eur J Appl Physiol. 2006;96(1):24-31. 16235068 10.1007/s00421-005-0061-0

[26] Ogawa M, Matsumoto T, Harada R, et al. Reliability and validity of quadriceps muscle thickness measurements in ultrasonography: a comparison with muscle mass and strength. Prog Rehabil Med. 2023;8:20230008. 36909302 10.2490/prm.20230008 PMC9998244

[27] Watanabe T, Murakami H, Fukuoka D, et al. Quantitative sonographic assessment of the quadriceps femoris muscle in healthy Japanese adults. J Ultrasound Med. 2017;36(7):1383-95. 28390140 10.7863/ultra.16.07054

[28] Andriopoulou M, Dimaki N, Kallistratos MS, et al. Skeletal muscle alterations and exercise intolerance in heart failure with preserved ejection fraction patients: ultrasonography assessment of diaphragm and quadriceps. Eur J Heart Fail. 2022;24(4):729-31. 35229401 10.1002/ejhf.2462

[29] Sato Y, Shiraishi H, Nakanishi N, et al. Clinical significance of rectus femoris diameter in heart failure patients. Heart Vessels. 2020;35(5):672-80. 31701229 10.1007/s00380-019-01534-7

[30] Kinugasa Y, Miyagi M, Sota T, et al. Dynapenia and diaphragm muscle dysfunction in patients with heart failure. Eur J Prev Cardiol. 2018;25(16):1785-6. 30080099 10.1177/2047487318793212

[31] Miyagi M, Kinugasa Y, Sota T, et al. Diaphragm muscle dysfunction in patients with heart failure. J Card Fail. 2018;24(4):209-16. 29289723 10.1016/j.cardfail.2017.12.004

[32] Fukumoto Y, Ikezoe T, Yamada Y, et al. Skeletal muscle quality assessed from echo intensity is associated with muscle strength of middle-aged and elderly persons. Eur J Appl Physiol. 2012;112(4):1519-25. 21847576 10.1007/s00421-011-2099-5

